# Assessing the evidence for shared genetic risks across psychiatric disorders and traits

**DOI:** 10.1017/S0033291717003440

**Published:** 2017-12-04

**Authors:** Joanna Martin, Mark J. Taylor, Paul Lichtenstein

**Affiliations:** 1 Department of Medical Epidemiology and Biostatistics, Karolinska Institutet, Stockholm, Sweden; 2 MRC Centre for Neuropsychiatric Genetics and Genomics, Cardiff University, Cardiff, UK

**Keywords:** genetic correlation, twin studies, genetics, pleiotropy, GWAS

## Abstract

Genetic influences play a significant role in risk for psychiatric disorders, prompting numerous endeavors to further understand their underlying genetic architecture. In this paper, we summarize and review evidence from traditional twin studies and more recent genome-wide molecular genetic analyses regarding two important issues that have proven particularly informative for psychiatric genetic research. First, emerging results are beginning to suggest that genetic risk factors for some (but not all) clinically diagnosed psychiatric disorders or extreme manifestations of psychiatric traits in the population share genetic risks with quantitative variation in milder traits of the same disorder throughout the general population. Second, there is now evidence for substantial sharing of genetic risks across different psychiatric disorders. This extends to the level of characteristic traits throughout the population, with which some clinical disorders also share genetic risks. In this review, we summarize and evaluate the evidence for these two issues, for a range of psychiatric disorders. We then critically appraise putative interpretations regarding the potential meaning of genetic correlation across psychiatric phenotypes. We highlight several new methods and studies which are already using these insights into the genetic architecture of psychiatric disorders to gain additional understanding regarding the underlying biology of these disorders. We conclude by outlining opportunities for future research in this area.

## Introduction

**Fig. 1.**
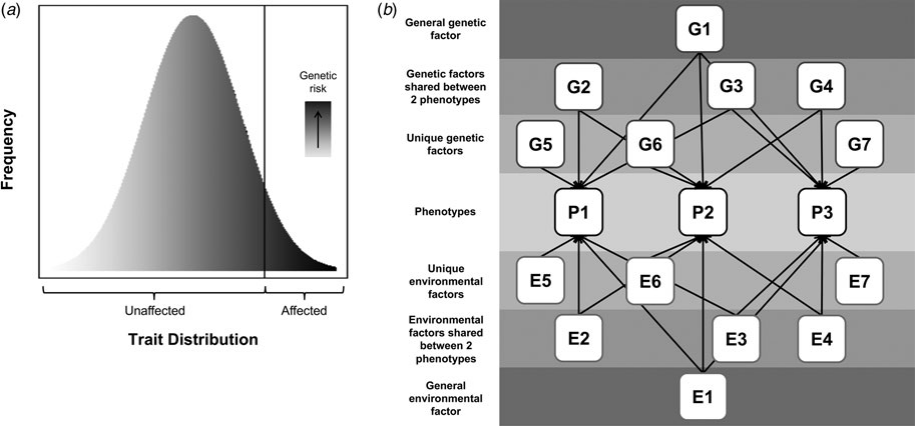
Hypothesized models of: (*a*) shared genetic risks across disorder and population trait variation, where the extreme end of a continuous distribution of a trait is associated with a continuous underlying genetic liability, and (*b*) shared genetic risks across different disorders, where squares labeled ‘P’ represent phenotypes, and squares labeled ‘G’ and ‘E’, represent genetic or environmental contributions, respectively, that can be shared or unique (indicated by the number of arrows pointing to phenotypes). All G factors are uncorrelated with one another and thus the entire genetic contribution to a phenotype can be modelled as the sum of the genetic factors contributing to it (e.g. for P1 this would be G1 + G2 + G3 + G5). The same is true for environmental factors (i.e. environmental contribution to P1 is E1 + E2 + E3 + E5). As an illustrative example, if P1 were ADHD, P2 were ASD, and P3 were MDD, then G1 represents any genetic variants that are shared between ADHD, ASD, and MDD; G2–G4 represents genetic variants shared between only two of these disorders (e.g. G2 would be genetic risk for ADHD and ASD but not MDD); and G5–G7 represent unique genetic risks (e.g. G5 is genetic risk that is unique to ADHD and not shared with either ASD or MDD). N.B. The shapes are not indicative of whether a variable is latent or measured.

Psychiatric disorders are relatively common in terms of lifetime prevalence and are associated with considerable distress and functional impairment (Whiteford *et al.* 2013). Understanding the etiology of these disorders is of critical importance to developing effective treatments and reducing suffering. There is strong evidence that these disorders are complex and partly genetic in origin, with twin study heritability estimates of 40–80% (Polderman *et al.* 2015). Environmental factors also contribute and possibly moderate genetic risk. This review will consider two important related hypotheses: that psychiatric disorders share genetic risks with variation in relevant population traits (illustrated in Fig. 1*a*) and that there are shared genetic contributions across different psychiatric phenotypes (illustrated in Fig. 1*b*).

The hypothesis that psychiatric disorders are extreme manifestations of continuously distributed population traits is not new [e.g. for a theoretical review see (Plomin *et al*. 2009)]. However, studies specifically testing whether categorical, clinical disorders share genetic risks with continuous variation in related sub-diagnostic traits in the population have been sparse until recently. A pressing matter that needs to be evaluated for specific psychiatric phenotypes, is the extent to which the current evidence supports this hypothesis. Recent years have also seen a dramatic increase in studies examining the related issue of shared genetic risks across *different* psychiatric disorders. Given the fast-growing body of research on this subject, the time is ripe to assess the strength of the evidence of shared risks for specific pairs of psychiatric phenotypes. In this review, we summarize and evaluate the evidence for the two hypotheses illustrated in Fig. 1, for a range of psychiatric phenotypes that have been extensively studied using both traditional twin and molecular genetic methods. We also discuss possible interpretations and implications for genetic research and clinical practice. Based on a non-exhaustive literature search, studies were included if they formally tested for shared genetic risks across a psychiatric disorder and traits related to the same disorder or across different psychiatric phenotypes (either defined as disorder or traits). Twin studies using DeFries-Fulker analysis were also included, although these studies do not directly test for genetic correlation; see additional discussion below.

## Overview of twin and molecular genetic methods

Most evidence concerning shared genetic risks within and across phenotypic constructs comes from twin studies and common variant genome-wide analyses. The twin design, which relies on comparison of identical (monozygotic) and non-identical (dizygotic) individuals, is commonly used to estimate the heritability of individual traits. Of particular relevance is the DeFries-Fulker analytic method, which estimates group heritability. Group heritability indicates the degree to which the mean difference between a proband group and the rest of a given sample is influenced by genetic factors. Significant group heritability indicates similar etiology for milder variation in continuous traits and more severe manifestations. An extension of the twin design, the bivariate twin model, allows one to estimate the degree of genetic correlation (*r*_g_) between two phenotypes. Complementarily, molecular genetic methods directly test for shared genetic risks across phenotypes. One method is the estimation of genetic correlation [e.g. using LDSC or GREML-GCTA (Yang *et al.* 2011; Bulik-Sullivan *et al.* 2015*a*)] from millions of common variants (single nucleotide polymorphisms; SNPs), for example using a case-control sample of one psychiatric disorder and another sample assessed for a relevant continuous trait or a different disorder. Such methods provide correlation estimates of the degree to which genetic risks are shared. However, practical limitations include a need for very large sample sizes and for some methods (e.g. GREML-GCTA), access to raw genotypes, limiting the application of these tools. A second approach uses a genome-wide association study (GWAS) ‘discovery’ sample to calculate polygenic risk scores (PRS) (Wray *et al.* 2014) for individuals in an independent ‘target’ sample. PRS for a phenotype of interest can be tested for association with another phenotype (e.g. another psychiatric disorder or trait variation) in the target sample, to establish whether there are shared genetic risks across phenotypes. Although studies using PRS methods can show direct evidence for shared genetic risks, typically modest effect sizes are observed (Wray *et al.* 2014), whereas molecular genetic studies that estimate genetic correlation provide a more precise assessment of the degree of shared genetic risks across phenotypes using different definitions.

It is important to note several differences in the meaning of results obtained from twin and molecular genetic analyses. For a more thorough review of different methods for estimation of univariate heritability and genetic correlation, please see Yang *et al.* (2017). In brief, the correlation estimates from twin studies capture all inherited genetic variants shared by monozygotic twins. These estimates are likely to be different and higher than those from SNP-based studies as the latter is only based on additive common variant effects tagged by genotyping arrays. However, the source of any shared genetic effects cannot be discerned from twin studies; effects may be driven by or limited to specific types of variants (e.g. rare mutations) but not to other classes of variants (e.g. SNPs). Genetic studies assessing multiple classes of variants are needed to determine the source of genetic correlations estimated using twin studies. It is also worth noting that evidence from common and rare variant studies regarding shared genetic risks between two phenotypes might not be consistent.

## Shared genetic risks across categorical disorders and population trait variation

Genetic studies have consistently demonstrated that thousands of common variants of small effect, as well as rare variants of larger effect, increase the risk for psychiatric disorders (Sullivan *et al*. 2012; Cross-Disorder Group of the PGC, 2013*a*; Davis *et al.* 2013; Robinson *et al*. 2015). This complex polygenic architecture supports a model where a quantitatively distributed liability (influenced by numerous genes) is associated with one or more continuous phenotypes that underlie the diagnostic distinction between cases and controls. According to such a model (Fig. 1*a*), genetic risks that contribute to clinical diagnoses will also influence variation in related quantitative traits in the general population. See Table 1 for a summary of studies that have addressed this hypothesis for specific psychiatric disorders.

**Table 1.** Summary of studies investigating shared genetic risks across disorders and trait variation

Disorder	Evidence from studies
ASD	*Twin studies*: persistently high group heritability across varying cut-offs; Genetic correlation of 0.70 between clinical diagnoses of ASD and autistic traits (Robinson *et al.* 2011; Lundström *et al.* 2012; Colvert *et al.* 2015) *LDSC*: ASD and population social-communication traits: rg = 0.27; replicated in independent clinical sample: rg = 0.30; genetic correlation is highest at age 8 years (rg = 0.34) and drops and is no longer significant for traits measured at ages 11 (0.16), 14 (0.21), and 17 (0.01) years (Robinson *et al.* 2016; St Pourcain *et al.* 2017) *PRS*: PRS derived using a clinical ASD discovery GWAS showed significant association with social-communication problems at age 8 years but not ages 11, 14, and 17 years in the general population (St Pourcain *et al.* 2017) and self-reported autistic traits in adults (in particular symptoms related to attention to detail, but also rigidity and childhood behaviors) (Bralten *et al.* 2017), although 1 smaller study does not find an association with autistic traits in children (Krapohl *et al.* 2016) *Other*: rare *de novo* loss-of-function and missense mutations associated with Vineland composite scores in ASD probands and unaffected siblings (Robinson *et al.* 2016)
ADHD	*Twin studies*: high group heritability from DeFries-Fulker analysis (Levy *et al.* 1997; Larsson *et al.* 2011; Greven *et al.* 2016) *LDSC*: ADHD and population traits of ADHD: rg = 0.96 (Middeldorp *et al.* 2016); replicated in a larger study: rg = 0.94 (Demontis *et al.* 2017). ADHD and traits of extraversion in the population: rg = 0.30 (Lo *et al.* 2016) *PRS*: multiple analyses of independent target samples find associations between clinically-defined ADHD PRS and population traits of ADHD and vice versa, although 1 smaller study does not find an association (Groen-Blokhuis *et al.* 2014; Martin *et al.* 2014*a*; Stergiakouli *et al.* 2015, 2017; Krapohl *et al.* 2016; Riglin *et al.* 2016; Brikell *et al.* 2017; Jansen *et al.* 2017)
ID	*Twin studies*: Cognitive abilities in the general population seem to share genetic risks with milder forms of ID (Spinath *et al.* 2004; Reichenberg *et al.* 2016) and extremely high IQ (Shakeshaft *et al.* 2015). There is some evidence, however, of discontinuity between cognitive abilities and severe forms of ID (Reichenberg *et al.* 2016) *Other*: Rare, likely pathogenic copy number variants (which are associated with developmental delay) are also associated with lower cognitive ability in the population (Männik *et al.* 2015; Kendall *et al.* 2016)
Anxiety disorders	*Twin studies*: extreme over-anxiety and specific fears, such as of animals, seem to share genetic risks with milder trait anxiety (Stevenson *et al*. 1992; Goldsmith & Lemery, 2000). Notably, the evidence is lacking on links between anxiety disorders and anxiety traits in samples that are closer in age to the typical age of onset for anxiety disorders
OCD	*Twin studies*: although heritable OCD traits are present throughout the general population (van Grootheest *et al.* 2005), no study has focused on the extreme presentation of these traits *PRS*: OCD PRS were associated with OCD traits in the general population (den Braber *et al.* 2016)
MDD	*Twin studies*: smaller studies yielded inconsistent findings; one study did not find significant group heritability, although a subsequent study of children and adolescents did (Rende *et al.* 1993; Eley, 1997) *PRS*: MDD PRS associated with depressive symptoms in elderly (Demirkan *et al.* 2011) but not with internalizing traits at ages 3–10 years (Jansen *et al.* 2017) *LDSC*: MDD and depressive symptoms in the population: rg = 0.91–1.00; MDD and traits of neuroticism in the population: rg = 0.56–0.70 (Direk *et al.* 2016; Lo *et al.* 2016; Major Depressive Disorder Working Group of the PGC *et al*. 2017)
SCZ & psychosis	*Twin studies*: Significant group heritability for severe and milder manifestations of adolescent psychotic experiences, suggesting genetic links between mild and severe psychotic experiences; however, the association with psychotic disorders such as SCZ is unclear from these studies (Ronald *et al.* 2014*b*; Zavos *et al.* 2014) *PRS*: no association of SCZ PRS and psychotic experiences in adolescents; evidence of association of SCZ PRS and adolescent negative schizophrenia-like symptoms as well as ‘thought problems’ at age 10 years; increased PRS in unaffected relatives and with increasing severity in probands (Zammit *et al.* 2013; Bigdeli *et al.* 2014; Sieradzka *et al.* 2014; Jones *et al.* 2016; Krapohl *et al.* 2016; Meier *et al.* 2016; Jansen *et al.* 2017)

ASD, autism spectrum disorder; ADHD, attention-deficit hyperactivity disorder; ID, intellectual disability; OCD, obsessive-compulsive disorder; MDD, major depressive disorder; SCZ, schizophrenia

Group heritability (implemented in DeFries-Fulker analysis) (DeFries & Fulker, 1985) refers to the degree to which genetic factors influence the mean difference between extreme groups and the rest of a sample; significant group heritability implies a genetic link between milder and more severe manifestations of a trait

Linkage disequilibrium score correlation (LDSC) (Bulik-Sullivan *et al.* 2015*a*, *b*) estimates the contribution of all SNPs from genome-wide data and indexes this as an estimate of SNP-heritability; which is different to twin heritability (Wray *et al.* 2014). This method can be applied to examine shared genetic risks between disorders and population traits to give an estimate of genetic correlation. Genome-wide association studies (GWAS) directly assess the independent association of many millions of common genetic variants (single nucleotide polymorphisms; SNPs) with a phenotype. Polygenic risk score (PRS) analysis, uses a GWAS ‘discovery’ sample to calculate genetic risk scores for individuals in an independent ‘target’ sample with genetic data; scores are derived by calculating the number of risk alleles weighted by the discovery effect size for each SNP and then summing these values for the set of SNPs, for each target individual (The International Schizophrenia Consortium, 2009). Regression analyses are used to test whether PRS for the discovery phenotype (e.g. clinical disorder) are associated with phenotypes of interest in the independent target sample (e.g. symptom variation in the population)

## Disorders with early onset

Twin studies have reported significant group heritability using several different definitions of ASD (Robinson *et al.* 2011; Lundström *et al.* 2012). One study employed a novel twin model to estimate the genetic correlation between ASD diagnoses and traits (*r*_g_ = 0.70) (Colvert *et al.* 2015). PRS studies show mixed results, with association between clinical ASD PRS with social-communication problems at age 8 but not later ages (St Pourcain *et al.* 2017), with self-assessed autistic traits in adults (Bralten *et al.* 2017) and null results in a third study (Krapohl *et al.* 2016). Modest, genetic correlation (*r*_g_ = 0.27–0.34) was estimated between clinical ASD and social-communication traits at age 8, with non-significant estimates at ages 11–17 years (Robinson *et al.* 2016; St Pourcain *et al.* 2017). The rate of rare *de novo* mutations was associated with autism-related behaviors not only in children with ASD but also in unaffected siblings (Robinson *et al.* 2016).

Twin studies of attention-deficit hyperactivity disorder (ADHD) traits have also revealed substantial group heritability for extreme scores on ADHD traits (Levy *et al.* 1997; Larsson *et al.* 2011), albeit extremely low ADHD scores are a potential exception (Greven *et al.* 2016). Multiple PRS analyses have demonstrated that genetic risk for clinically-diagnosed ADHD is shared with ADHD traits assessed between ages 3 and 17 years (Groen-Blokhuis *et al.* 2014; Martin *et al.* 2014*a*; Stergiakouli *et al.* 2015, 2017; Riglin *et al.* 2016; Brikell *et al.* 2017; Jansen *et al.* 2017). Estimates of genetic correlation between ADHD diagnosis and traits are very high (*r*_g_ = 0.94–0.96) (Middeldorp *et al.* 2016; Demontis *et al.* 2017), with a moderate genetic correlation (*r*_g_ = 0.30) between ADHD diagnosis and extraversion traits in the population (Lo *et al.* 2016).

Cognitive abilities display a similar pattern of significant group heritability in studies of mild intellectual disability (ID) (Spinath *et al.* 2004), different quantiles of reading assessments (Logan *et al.* 2012), and high levels of intelligence (Shakeshaft *et al.* 2015). However, severe ID appears to be an exception to this pattern (Reichenberg *et al.* 2016). Molecular genetic studies of ID have focused on very rare mutations (Girirajan *et al.* 2011; The Deciphering Developmental Disorders Study, 2014) and there is some evidence that rare, likely pathogenic copy number variants (CNVs) are associated with poor performance on cognitive tasks in the population (Männik *et al*. 2015; Kendall *et al.* 2016). Studies assessing the degree of shared common variants between ID and cognition in the population are lacking.

Converging evidence from twin and molecular genetic methods so far shows reasonably strong support for certain child-onset neurodevelopmental disorders (i.e. ADHD, ASD, and mild ID) as the extreme ends of continuous distributions of population traits.

## Disorders with onset in adolescence and adulthood

There is a lack of studies testing for shared genetic risks across disorder and traits for anxiety disorders and obsessive-compulsive disorder (OCD). Although twin studies have established the heritability of anxiety traits, only two studies reported significant group heritability for anxiety disorders (Stevenson *et al*. 1992; Goldsmith & Lemery, 2000). Twin studies of OCD indicate that traits characteristic of OCD are heritable and present throughout the population (van Grootheest *et al.* 2005), although no twin studies have tested whether extreme OCD traits share genetic risks with milder traits. One recent study found associations between OCD PRS and continuously-distributed obsessive-compulsive traits in the population (den Braber *et al.* 2016).

Twin studies of group heritability for depressive traits have found mixed results (Rende *et al.* 1993; Eley, 1997). Shared genetic influences across major depressive disorder (MDD) and depressive traits have been reported in an elderly population using PRS analysis (Demirkan *et al.* 2011) but not in a childhood sample assessing internalizing traits at ages 3–10 years (Jansen *et al.* 2017). Recent common variant analyses showed very high genetic correlation (*r*_g_ = 0.91–1.00) between MDD and depressive symptoms (Direk *et al.* 2016; Anttila *et al.* 2017; Major Depressive Disorder Working Group of the PGC *et al*. 2017) and moderate correlation between MDD and personality measures, notably neuroticism (*r*_g_ = 0.56–0.74), in the general population (Lo *et al.* 2016; Major Depressive Disorder Working Group of the PGC *et al*. 2017).

The genetic evidence for a continuous spectrum of psychosis in the population is more complex. Psychotic experiences (e.g. paranoia and hallucinations) show low-to-moderate heritability (15–59%), with significant group heritability implying a genetic link between mild and severe psychotic experiences (Zavos *et al.* 2014). However, it is unclear from twin studies whether psychotic experiences are related to schizophrenia. Findings from PRS studies are mixed, with several studies finding no association of schizophrenia or bipolar disorder (BD) PRS with adolescent psychotic experiences (Sieradzka *et al.* 2014; Krapohl *et al.* 2016), others reporting an association in the opposite direction to that expected (Zammit *et al.* 2013) and others finding associations between schizophrenia PRS and adolescent negative symptoms (e.g. apathy or lack of energy) related to schizophrenia (Jones *et al.* 2016) and ‘thought problems’ at age 10 (Jansen *et al.* 2017). Schizophrenia PRS are higher in unaffected relatives of schizophrenia probands compared with controls (Bigdeli *et al.* 2014) and in individuals with more strictly defined schizophrenia, in terms of chronicity or severity of disorder (Meier *et al.* 2016).

Evidence for shared genetic risks across disorders and traits is limited for adolescent- and adult-onset psychiatric disorders. Preliminary supporting evidence is seen for OCD and MDD. The picture is quite complex for schizophrenia and there is insufficient evidence to conclude whether anxiety disorders share genetic risks with related population traits.

## Limitations and interpretation

There are several limitations of existing studies and important issues that have not been sufficiently addressed. First, many twin studies use percentile-based cut-offs to identify probands, rather than using clinical diagnoses. Second, twin studies have largely employed DeFries-Fulker analysis, which does not directly estimate genetic correlation between psychiatric disorders and related traits; rather, significant group heritability suggests a link between extreme values of a trait and variation in the trait. Direct estimation of the genetic correlation, as done for ASD (Colvert *et al.* 2015), would likely be informative in future twin research.

Although analyses of population traits do not include many individuals who have psychiatric diagnoses, it is important to determine whether associations persist when such individuals are excluded. If not, this might suggest that any association signal is driven by extreme cases and not continuous variation in the trait of interest. Another important issue is the strength of any observed genetic correlations. It is entirely likely that even if there is some degree of shared genetic risk between a disorder and related traits, this will be partial and unique genetic effects will also contribute [e.g. as may be the case with ASD and social-communication traits, given somewhat modest genetic correlations (Robinson *et al.* 2016)].

Given that most psychiatric disorders consist of multiple domains, another challenge is identifying whether relevant population traits show different degrees of shared genetic risk with a given psychiatric disorder, as seems to be the case for schizophrenia genetic risk in relation to psychotic experiences and negative symptoms in the population (Jones *et al.* 2016). Another difficulty with analyzing continuously distributed psychiatric traits is capturing the full spectrum of a relevant behavior, as most measurement instruments are optimized for detecting difficulties not abilities, thereby resulting in highly zero-inflated and skewed distributions that often violate modeling assumptions. It is unknown whether normalizing such scores through transformations or by regressing out covariates and rank-transforming a variable is an optimal solution and such methods may introduce technical artifacts (Pain *et al*. 2017). Skewed variables need to be analysed using models that appropriately account for non-normal distributions of data. Ideally, measures that better capture the full variability of behavioral phenotypes are also needed.

We suggest that the assessment of the degree to which a heritable disorder can be considered as an extreme manifestation of population traits should include the following investigations: estimation of the heritability of relevant population traits, estimation of genetic correlation between the disorder and traits, and sensitivity analyses to determine whether any correlation is explained entirely by inclusion of individuals scoring at the extreme end of the trait distribution.

## Shared genetic risks across different psychiatric phenotypes

Whilst the degree to which many specific psychiatric disorders share genetic risk with related population traits is yet to be determined, there is much more evidence regarding shared genetic risks across different disorders. Below we consider the strength of the evidence examining this hypothesis, as illustrated in Fig. 1*b*. See Table 2 for a summary. It is important to note that many studies have examined shared genetic risk between one psychiatric disorder and population traits related to another phenotype, thereby providing additional, albeit indirect, evidence for sharing of genetic risks across psychiatric disorders and continuous traits.

**Table 2.** Summary of studies investigating shared genetic risks across disorders

Disorder	ASD	ADHD	ID	SCZ	BD	MDD	AXD	AN&ED	OCD
ADHD	*Twin rg*: 0.54–0.87 (Reiersen *et al.* 2008; Ronald *et al.* 2008; Lichtenstein *et al.* 2010) *SNP rg*: ns (Cross-Disorder Group of the PGC, 2013*a*; Bulik-Sullivan *et al.* 2015*a*; Anttila *et al.* 2017) *PRS*: mixed evidence (Cross-Disorder Group of the PGC, 2013*b*; Martin *et al.* 2014*a*; Krapohl *et al.* 2016; Brikell *et al.* 2017; Jansen *et al.* 2017) *Other*: Overlap of CNV loci (Lionel *et al.* 2011; Williams *et al.* 2012)								
ID	*Twin rg*: 0.04–0.71 (disorder) & −0.27 (traits) (Hoekstra *et al.* 2009, 2010; Lichtenstein *et al.* 2010) *PRS*: positive association (Clarke *et al.* 2016) *SNP rg*: 0.21–0.38 between ASD and general cognition (Anttila *et al.* 2017; Sniekers *et al.* 2017) *Other*: Overlap of CNV loci & genes hit by rare loss-of-function mutations (Guilmatre *et al.* 2009; Pescosolido & Gamsiz, 2013; De Rubeis *et al.* 2014; Iossifov *et al.* 2014; Samocha *et al.* 2014)	*Twin rg*: −0.16 to −0.41 (Greven *et al.* 2011, 2014) *PRS*: negative association with IQ, positive association with learning difficulties (Martin *et al.* 2014*b*; Clarke *et al.* 2016; Brikell *et al.* 2017) *SNP rg*: −0.27 to −0.41 between ADHD and general cognition (Demontis *et al.* 2017; Sniekers *et al.* 2017) *Other*: Overlap of CNV loci (Lionel *et al.* 2011; Williams *et al.* 2012)							
SCZ	*SNP rg*: 0.16–0.23 (Cross-Disorder Group of the PGC, 2013*a*; Bulik-Sullivan *et al.* 2015*a*; Anttila *et al.* 2017; The ASD Working Group of The PGC, 2017) *PRS*: association with disorder & ASD traits (Cross-Disorder Group of the PGC, 2013*b*; Krapohl *et al.* 2016; St Pourcain *et al.* 2017) *Other*: Overlap of CNV loci (Guilmatre *et al.* 2009)	*SNP rg*: 0.22–0.23 (Bulik-Sullivan *et al.* 2015*a*; Anttila *et al.* 2017) *PRS*: mixed evidence for ADHD disorder (Cross-Disorder Group of the PGC, 2013*b*; Hamshere *et al.* 2013); mixed for population traits of ADHD (Krapohl *et al.* 2016; Jansen *et al.* 2017; Nivard *et al.* 2017) *Other*: Overlap of CNV loci (Lionel *et al.* 2011; Williams *et al.* 2012)	*Twin rg*: 0.15–0.22 between communication impairment and adolescent psychotic-like experiences (Cederlöf *et al.* 2014*a*) *SNP rg*: −0.38 (performance IQ); −0.07 (verbal IQ); −0.20 (general cognition) (Hubbard *et al.* 2016; Anttila *et al.* 2017; Sniekers *et al.* 2017) *PRS*: negative association with cognition (McIntosh *et al*. 2013; Lencz *et al.* 2014; Hagenaars *et al.* 2016; Hubbard *et al.* 2016) *Other*: Overlap of CNV loci (Guilmatre *et al.* 2009)						
BD	*Twin rg*: 0.24 (Song *et al.* 2015) *SNP rg*: ns (Cross-Disorder Group of the PGC, 2013*a*; Bulik-Sullivan *et al.* 2015*a*; Anttila *et al.* 2017) *PRS*: positive association with disorder; mixed for ASD traits (Cross-Disorder Group of the PGC, 2013*b*; Krapohl *et al.* 2016)	*Twin rg*: 0.33 (Song *et al.* 2015) *SNP rg*: 0.26–0.71 (Bulik-Sullivan *et al.* 2015*a*; van Hulzen *et al.* 2016; Anttila *et al.* 2017) *PRS*: ns (Cross-Disorder Group of the PGC, 2013*b*; Hamshere *et al.* 2013; Krapohl *et al.* 2016; Jansen *et al.* 2017)	*Twin rg*: 0.30 between communication impairment and juvenile mania symptoms (Cederlöf *et al.* 2014*b*) *SNP rg*: ns (general cognition) (Anttila *et al.* 2017; Sniekers *et al.* 2017)	*Twin rg*: 0.28–0.60; 49–68% shared liability (Cardno *et al.* 2002; Lichtenstein *et al.* 2009; Song *et al.* 2015) *SNP rg*: 0.68–0.79 (Cross-Disorder Group of the PGC, 2013*a*; Bulik-Sullivan *et al.* 2015*a*; Anttila *et al.* 2017) *PRS*: consistent positive association (disorder) (The International Schizophrenia Consortium, 2009; Cross-Disorder Group of the PGC, 2013*b*)					
MDD	*Twin rg*: 0.17–0.19 (Hallett *et al.* 2010) *SNP rg*: 0.44 between disorders; ns between ASD and depressive symptoms (Cross-Disorder Group of the PGC, 2013*a*; Bulik-Sullivan *et al.* 2015*a*; Anttila *et al.* 2017; Major Depressive Disorder Working Group of the PGC *et al*. 2017) *PRS*: ns (Cross-Disorder Group of the PGC, 2013*b*; Krapohl *et al.* 2016; Jansen *et al.* 2017)	*Twin rg*: 0.34–0.77 (Cole *et al.* 2009; Chen *et al.* 2016; Rydell *et al*. 2017) *SNP rg*: 0.32–0.52 between disorders; 0.40–0.45 between ADHD and depressive symptoms (Cross-Disorder Group of the PGC, 2013*a*; Bulik-Sullivan *et al.* 2015*a*; Anttila *et al.* 2017; Demontis *et al.* 2017; Major Depressive Disorder Working Group of the PGC *et al*. 2017) *PRS*: ns (Cross-Disorder Group of the PGC, 2013*b*; Krapohl *et al.* 2016; Jansen *et al.* 2017)	*SNP rg*: − 0.27 between depressive symptoms and general cognition; ns for MDD (Anttila *et al.* 2017; Sniekers *et al.* 2017)	*Twin rg*: 0.72–0.78 (traits) (Zavos *et al.* 2016) *SNP rg*: 0.34–0.51 between disorders; 0.30 between depressive symptoms and SCZ (Cross-Disorder Group of the PGC, 2013*a*; Bulik-Sullivan *et al.* 2015*a*; Anttila *et al.* 2017; Major Depressive Disorder Working Group of the PGC *et al*. 2017) *PRS*: positive association (disorder) (Cross-Disorder Group of the PGC, 2013*b*); mixed evidence (depressive or internalizing traits) (Jones *et al.* 2016; Nivard *et al.* 2017)	*Twin rg*: 0.35 (Song *et al.* 2015) *SNP rg*: 0.32–0.48 between disorders; 0.28 between BD and depressive symptoms (Cross-Disorder Group of the PGC, 2013*a*; Bulik-Sullivan *et al.* 2015*a*; Anttila *et al.* 2017; Major Depressive Disorder Working Group of the PGC *et al*. 2017) *PRS*: positive association (disorder) (Cross-Disorder Group of the PGC, 2013*b*); ns (internalizing traits) (Jansen *et al.* 2017)				
AXD	*Twin rg*: 0.17–0.19 (Hallett *et al.* 2010) *SNP rg*: ns (Anttila *et al.* 2017) *PRS*: ns (Krapohl *et al.* 2016; Jansen *et al.* 2017)	*Twin rg*: 0.45–0.58 (Michelini *et al.* 2015; Chen *et al.* 2016) *SNP rg*: ns (Anttila *et al.* 2017) *PRS*: ns (Krapohl *et al.* 2016)	*SNP rg*: ns (general cognition) (Anttila *et al.* 2017; Sniekers *et al.* 2017)	*SNP rg*: ns (Otowa *et al.* 2016; Anttila *et al.* 2017) *PRS*: ns (across disorders); replicated evidence for anxiety symptoms, though 1 study found no effect (Jones *et al.* 2016; Krapohl *et al.* 2016; Nivard *et al.* 2017)	*Twin rg*: 0.23 (Song *et al.* 2015) *SNP rg*: ns (Otowa *et al.* 2016; Anttila *et al.* 2017) *PRS*: Mixed (disorder) (Otowa *et al.* 2016); ns (traits) (Krapohl *et al.* 2016; Jansen *et al.* 2017)	*Twin rg*: 0.70–1.00 (Roy *et al.* 1995; Thapar & McGuffin, 1997; Kendler *et al.* 2007; Mosing *et al.* 2009; Demirkan *et al.* 2011) *SNP rg*: 0.68–0.80 between disorders; 0.82 between AXD and depressive symptoms (Otowa *et al.* 2016; Anttila *et al.* 2017; Major Depressive Disorder Working Group of the PGC *et al*. 2017) *PRS*: positive association (disorder) (Otowa *et al.* 2016); mixed (traits) (Krapohl *et al.* 2016; Jansen *et al.* 2017)			
AN&ED	*SNP rg*: ns (Bulik-Sullivan *et al.* 2015*a*; Anttila *et al.* 2017)	*SNP rg*: ns (Bulik-Sullivan *et al.* 2015*a*; Anttila *et al.* 2017)	*SNP rg*: ns (general cognition) (Anttila *et al.* 2017; Sniekers *et al.* 2017)	*SNP rg*: 0.19–0.22 (Bulik-Sullivan *et al.* 2015*a*; Anttila *et al.* 2017)	*SNP rg*: ns (Bulik-Sullivan *et al.* 2015*a*; Anttila *et al.* 2017)	*Twin rg*: 0.70 (Slane *et al.* 2011) *SNP rg*: 0.13 between disorders; ns between AN & depressive symptoms (Bulik-Sullivan *et al.* 2015*a*; Anttila *et al.* 2017; Major Depressive Disorder Working Group of the PGC, *et al.* 2017)	*SNP rg*: ns (Anttila *et al.* 2017)		
OCD	*SNP rg*: ns (Anttila *et al.* 2017)	*SNP rg*: ns (Anttila *et al.* 2017) *Other*: Heritable latent factor underlying ADHD, OCD, and tics (Pinto *et al.* 2016)	*SNP rg*: ns (general cognition) (Anttila *et al.* 2017)	*SNP rg*: 0.33 (Anttila *et al.* 2017)	*SNP rg*: 0.31 (Anttila *et al.* 2017)	*Twin rg*: 0.71–0.86 (traits) (Bolhuis *et al.* 2014) *SNP rg*: 0.23 between disorders; ns between OCD & depressive symptoms (Anttila *et al.* 2017)	*SNP rg*: ns (Anttila *et al.* 2017) *Other*: Common genetic factor underlying OCD and anxiety symptoms (López-Solà *et al.* 2016)	*Twin rg*: 0.52 (Cederlöf *et al.* 2015) *SNP rg*: 0.52 (Anttila *et al.* 2017)	
TS	*Twin rg*: 0.60 (Lichtenstein *et al.* 2010) *SNP rg*: ns (Anttila *et al.* 2017)	*Twin rg*: 1.00 (Lichtenstein *et al.* 2010) *SNP rg*: ns (Anttila *et al.* 2017) *Other*: Heritable latent factor underlying ADHD, OCD, and tics (Pinto *et al.* 2016)	*SNP rg*: ns (general cognition)(Anttila *et al.* 2017)	*SNP rg*: ns (Anttila *et al.* 2017)	*SNP rg*: ns (Anttila *et al.* 2017)	*SNP rg*: 0.21 between disorders; ns between TS & depressive symptoms (Anttila *et al.* 2017)	*SNP rg*: ns (Anttila *et al.* 2017)	*SNP rg*: ns (Anttila *et al.* 2017)	*SNP rg*: 0.41–0.43 (Davis *et al.* 2013; Anttila *et al.* 2017) *Other*: Heritable latent factor underlying ADHD, OCD, and tics (Pinto *et al.* 2016)

ADHD, attention-deficit hyperactivity disorder; AN&ED, anorexia nervosa and other eating disorders; ASD, autism spectrum disorder; AXD, anxiety disorders; BD, bipolar disorder; ID, intellectual disability; MDD, major depressive disorder; OCD, obsessive-compulsive disorder; SCZ, schizophrenia; TS, Tourette's syndrome and other tic disorders; SNP, single nucleotide polymorphism; CNV, copy number variant; PRS, polygenic risk score analysis; ns, non-significant estimates based on published studies.

Twin rg is the correlation between the additive genetic variance components from twin studies. Note that the ‘twin rg’ in Lichtenstein *et al*. (2009) & Song *et al*. (2015) are estimated from family studies but with a similar approach as in twin studies. SNP rg: is the estimated genetic correlation from genome-wide association studies using LDSC (linkage disequilibrium score correlation) or GCTA (genome-wide complex trait analysis). Only results estimated to be nominally significantly different from zero (*p* < 0.05) are presented. For a more detailed explanation of the methods, please refer to the caption of Table 1. The GREML-GCTA method (genetic relatedness estimation through maximum likelihood using the GCTA software) (Yang *et al.* 2011; Lee *et al.* 2012) is conceptually similar to LDSC; it is used to estimate the contribution of all SNPs from genome-wide data (SNP-heritability) and can be applied to examine shared genetic risks between disorders and population traits to give an estimate of genetic correlation.

## Disorders with early onset

Twin studies of neuropsychiatric diagnoses and childhood traits consistently show significant genetic correlations. Associations have been seen between ADHD inattentive symptoms and difficulties in reading and mathematics (Greven *et al.* 2011, 2014; Wadsworth *et al.* 2015), categorically and continuously defined ADHD and ASD (Reiersen *et al.* 2008; Ronald *et al.* 2008, 2014*a*; Lichtenstein *et al.* 2010; Taylor *et al.* 2012), and ASD with learning difficulties and tics (which are associated with Tourette's syndrome) (Lichtenstein *et al.* 2010). However, two other twin studies of ASD and intellectual ability have reported low genetic correlations, although this might have been related to measurement differences (Hoekstra *et al.* 2009, 2010).

Analyses of common genetic variants so far have not confirmed the genetic correlation between ADHD and ASD observed in twin studies (Cross-Disorder Group of the PGC, 2013*a*, *b*; Bulik-Sullivan *et al.* 2015*a*; Anttila *et al.* 2017; Jansen *et al.* 2017). Clinical ADHD shares some genetic risk with social-communication traits (Martin *et al.* 2014*a*) and other neurodevelopmental and externalizing traits that make up a general factor of childhood psychopathology (Brikell *et al.* 2017). Clinical ADHD shares genetic risk with lower cognitive abilities in children and adults in the general population (Martin *et al.* 2014*b*; Clarke *et al.* 2016; Stergiakouli *et al.* 2016; Anttila *et al.* 2017; Demontis *et al.* 2017; Riglin *et al.* 2017; Sniekers *et al.* 2017). In ASD, there is a positive genetic correlation with common variants associated with cognitive ability, suggesting that these variants operate differently to common risk variants for other psychiatric phenotypes and to rare variants in the context of ASD (Clarke *et al.* 2016; Robinson *et al.* 2016; Anttila *et al.* 2017; Sniekers *et al.* 2017; Weiner *et al.* 2017). With regard to rare variants, studies of CNVs have implicated the same genomic regions in multiple disorders, including ASD, ID, and ADHD (Guilmatre *et al.* 2009; Sebat *et al*. 2009; Pinto *et al.* 2010; Williams *et al.* 2010, 2012; Cooper *et al.* 2011; Lionel *et al.* 2011; Sanders *et al.* 2011; Pescosolido & Gamsiz, 2013). Recent large exome sequencing studies have identified the first robust rare *de novo* protein-truncating mutations (variants which disrupt protein formation and are likely highly deleterious) associated with ASD, with many of the same genes found to harbor *de novo* mutations linked to ID (De Rubeis *et al.* 2014; Iossifov *et al.* 2014; Samocha *et al.* 2014; The Deciphering Developmental Disorders Study, 2014).

Twin and molecular studies have yielded some consistent findings, but larger genetic studies are needed to further understand the degree and source of shared genetic risks in these early-onset disorders. The association between ASD and ID is particularly complex, with shared risk for these phenotypes seen at the level of rare risk variants but a positive association seen for common variants; indeed these mixed genetic results may partly explain the low genetic correlations between these phenotypes in twin studies (Hoekstra *et al.* 2009, 2010).

## Disorders with onset in adolescence and adulthood

Twin studies have found substantial evidence of genetic correlations across schizophrenia and BD (Cardno *et al.* 2002; Lichtenstein *et al.* 2009), BD and MDD (Song *et al.* 2015), anxiety disorder subtypes (Mosing *et al.* 2009), specific anxiety disorders and MDD (Roy *et al.* 1995; Kendler *et al.* 2007; Mosing *et al.* 2009), traits of anxiety and depressive symptoms (Thapar & McGuffin, 1997), MDD and psychotic experiences in adolescence (Zavos *et al.* 2016), depressive symptoms and disordered eating scores (Slane *et al*. 2011), OCD and MDD (Bolhuis *et al.* 2014), and OCD with anxiety-related behaviors and anorexia nervosa (AN) (Cederlöf *et al.* 2015; López-Solà *et al.* 2016).

GWAS of adult psychiatric disorders have confirmed that common genetic variants associated with one disorder also play an important role in other disorders. Recent analyses using multiple genome-wide methods report shared genetic risks across schizophrenia, BD, MDD, and OCD, across schizophrenia, AN and OCD, and between MDD with anxiety disorders and AN (Cross-Disorder Group of the PGC, 2013*a*, *b*; Bulik-Sullivan *et al.* 2015*a*; Anttila *et al.* 2017; Major Depressive Disorder Working Group of the PGC *et al*. 2017). Shared genetic risks are seen across different anxiety disorders (generalized anxiety disorder, panic disorder and phobias) and with MDD, though not with BD or schizophrenia (Otowa *et al.* 2016). General population studies of schizophrenia PRS report associations with anxiety symptoms, with mixed evidence for association with depressive symptoms between ages 7 and 15 (Jones *et al.* 2016; Jansen *et al.* 2017; Nivard *et al.* 2017). MDD PRS were also associated with anxiety symptoms in an elderly population sample (Demirkan *et al.* 2011). Thus, there is evidence that a considerable degree of genetic influences are shared across multiple phenotypes, assessed categorically or continuously.

## Shared genetic risks across child- and adult-onset disorders

Childhood-onset disorders and disorders with an onset typically in adolescence or adulthood also share genetic risks. For example, twin studies find that early-onset-neurodevelopmental disorders share genetic risk with anxiety (Hallett *et al.* 2010; Michelini *et al.* 2015; Chen *et al.* 2016), MDD (Cole *et al.* 2009; Lundström *et al.* 2011), affective problems (Rydell *et al*. 2017), and OCD (Pinto *et al.* 2016). In a study of specific intellectual domains, problems with communication shared a modest degree of genetic risk with adolescent hallucinations and mania (Cederlöf *et al.* 2014*b*). Molecular genetic studies have reported genetic correlations between both ADHD and ASD with MDD, schizophrenia and BD (Cross-Disorder Group of the PGC, 2013*a*, *b*; Bulik-Sullivan *et al.* 2015*a*; van Hulzen *et al.* 2016; Anttila *et al.* 2017; Demontis *et al.* 2017; Major Depressive Disorder Working Group of the PGC *et al*. 2017; The ASD Working Group of The PGC, 2017). Tourette's syndrome shares genetic risks with OCD and MDD (Davis *et al.* 2013; Anttila *et al.* 2017). Genetic risk for schizophrenia is associated with numerous traits assessed across ages 3–15 years, including ADHD, aggression, irritability, language, and social abilities (Jansen *et al.* 2017; Nivard *et al.* 2017; Riglin *et al.* 2017). BD and MDD PRS were not found to be associated with early life (age 3–10 years) internalizing and externalizing problems (Jansen *et al.* 2017).

CNV loci implicated in children with ADHD, ASD, and ID have also been associated with schizophrenia (The International Schizophrenia Consortium, 2008; Guilmatre *et al.* 2009; Sebat *et al*. 2009; Williams *et al.* 2010, 2012; Lionel *et al.* 2011; Pescosolido & Gamsiz, 2013). Schizophrenia shares genetic risks with cognitive measures throughout the lifespan (McIntosh *et al*. 2013; Lencz *et al.* 2014; Hagenaars *et al.* 2016; Hubbard *et al.* 2016; Krapohl *et al.* 2016). General cognitive ability shows negative genetic correlations with schizophrenia and depressive symptoms, though not with BD, anxiety disorder, MDD, OCD or AN (Anttila *et al.* 2017; Sniekers *et al.* 2017). Genetic correlations across several psychiatric disorders and personality measures have also been reported (Lo *et al.* 2016; Anttila *et al.* 2017). Psychiatric phenotypes also more broadly share genetic contributions with other human complex traits, for example genetic risk for ADHD is shared with behavioral traits (e.g. smoking), brain- (e.g. migraine) and non-brain-based diseases (e.g. type-2-diabetes) and traits (e.g. body mass index) (Anttila *et al.* 2017; Demontis *et al.* 2017). A wider review is beyond the scope of this paper.

In summary, studies indicate that a considerable degree of genetic influences on particular disorders are shared with at least one other disorder, regardless of whether one focuses on childhood- or adulthood-onset conditions. It has been hypothesized that a single ‘general genetic factor’ underlies multiple psychiatric phenotypes (Lahey *et al.* 2012; Caspi *et al.* 2014). Two twin studies supported this model, with a latent genetic factor accounting for 31% of variance in neurodevelopmental symptoms in a population-based sample (Pettersson *et al.* 2013) and 10–36% of disorder liability across multiple clinical psychiatric diagnoses (Pettersson *et al*. 2015). A recent study further confirmed that common genetic risk variants contribute to this general factor, with an estimated SNP-heritability of approximately 0.38 (Neumann *et al.* 2016). As illustrated in Fig. 1*b*, the situation is likely to be even more complex, with not only a general genetic factor predisposing to multiple phenotypes but also disorder-specific genetic factors as well as genetic factors relevant only to specific pairs of disorders. Similarly, environmental factors could also be shared or unique and more complex effects, such as gene-environment interactions, could also exist.

Although several of the pairs of psychiatric disorders assessed using GWAS data do not show significant genetic correlations, some of the studies were relatively small and are likely to be underpowered. Notably, genetic correlations are present regardless of whether psychiatric phenotypes are conceptualized continuously or dichotomously, thus providing additional, albeit indirect, support for shared genetic risk across these disorders and related traits.

## Interpreting the meaning of genetic correlations

**Fig. 2.**
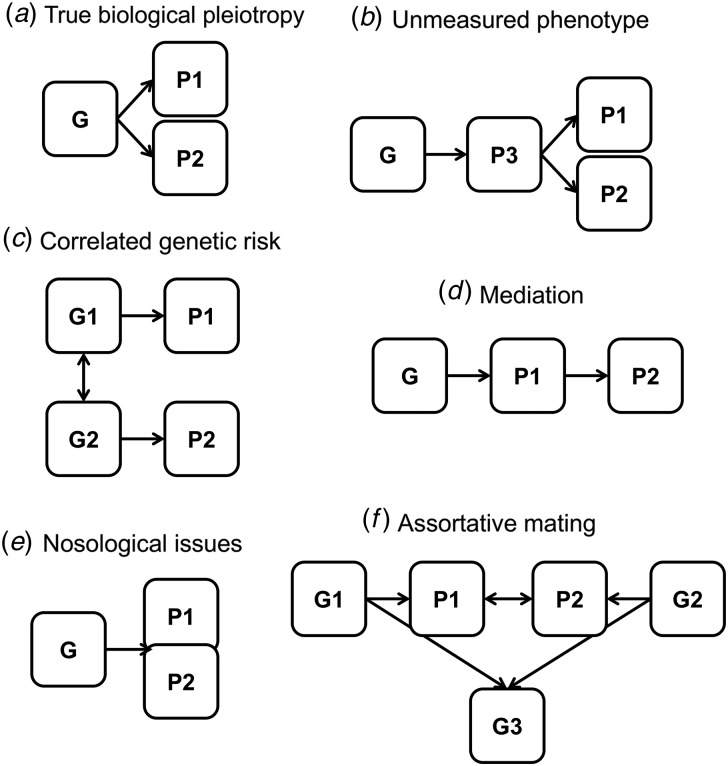
Potential interpretations of genetic correlation across phenotypes: (*a*) true biological pleiotropy, where the same genetic risk variant is causally associated with two phenotypes; (*b*) unmeasured phenotype, where a third phenotype is on the causal pathway between genetic risk and the outcome phenotypes of interest; (*c*) correlated genetic risk, where different genetic risk variants that are highly correlated are causally associated with each phenotype; (*d*) mediation, where a genetic risk variant only acts on one of the phenotypes, which in turn influences a second phenotype; (*e*) Nosological issues, which blur the distinction between phenotypes, for example comorbidity, ascertainment bias, heterogeneity or diagnostic misclassification; (*f*) assortative mating, where individuals with the two phenotypes of interest are more likely to mate than expected at random, thereby leading to clustering of genetic risk for both phenotypes in the offspring. N.B. The shapes are not indicative of whether a variable is latent or measured.

The interpretation of what genetic correlations mean is complex, with a number of possibilities, some of which are not mutually exclusive. One possibility (Fig. 2*a*) is that of true biological pleiotropy, where the same risk variants (or variants within the same gene) are directly, causally impacting on multiple phenotypes, albeit possibly through separate biological pathways. Alternatively, the same genetic risk variants could be causally affecting a third, unmeasured phenotype which lies on the pathway between risk variants and measured phenotypes (Fig. 2*b*). A third possibility (Fig. 2*c*) is that observed genetic correlations are actually capturing different risk variants that are highly correlated but are acting through different mechanisms. For example, even though the same CNV loci have been implicated in multiple disorders (Guilmatre *et al.* 2009; Lionel *et al.* 2011; Williams *et al.* 2012; Pescosolido & Gamsiz, 2013), different variants within these large loci might be associated with different phenotypes. Given that such large, rare variants are also shared by monozygotic twins, this could also influence estimates of genetic correlations based on twin studies. A fourth possibility (Fig. 2*d*) is that one phenotype mediates the association between genetic risk and a second phenotype and there is no direct causal relationship between the risk variant and this second phenotype. For example, it has been proposed that the genetic correlation between MDD and depressive symptoms in the population could be accounted for by shared genetic risk with low levels of subjective well-being (Direk *et al.* 2016).

Several nosological issues (Fig. 2*e*) may also explain genetic correlations to an extent. Comorbidity across disorders (e.g. anxiety and MDD) is frequently observed and certain symptom domains show similarities [e.g. manic (BD) or hyperactive (ADHD) symptoms]. Specific symptoms also overlap directly across disorders (e.g. concentration problems in ADHD, MDD or anxiety) and such overlap may largely account for comorbidity [e.g. anxiety and MDD (Cramer *et al.* 2010)]. Such phenotypic overlap could inflate genetic correlation estimates. Within-disorder heterogeneity could also induce an overall correlation across two phenotypes, when only a sub-group of individuals with one disorder (who may have a specific clinical profile) show genetic correlation with individuals with another phenotype. Another possibility is that of diagnostic misclassification or changes in meeting diagnostic criteria over time (e.g. individuals who are diagnosed with MDD but later develop manic features, leading to a diagnosis of BD). Given the similar diagnostic features across different disorders, accurate diagnosis is difficult. Fortunately, diagnostic changes over time can be taken into consideration using epidemiological family study designs (Song *et al.* 2015). Simulations show that a 10% rate in misclassification can inflate estimates of genetic correlation (Wray *et al*. 2012). However, very high degrees of such misclassification would be required to fully account for the observed genetic correlations across psychiatric phenotypes (Anttila *et al.* 2017). Such issues related to phenotype definition remain to be resolved as the underlying biology of psychiatric disorders is better understood. For now, careful ascertainment and better measurement of frequently co-occurring disorder-level and sub-threshold phenotypes is required.

Another possibility for interpreting observed genetic correlations between psychiatric disorders is that they arise through assortative mating (Fig. 2*f*). There are substantial effects of assortative mating both within and across multiple psychiatric disorders (Nordsletten *et al.* 2016). Such assortative mating across disorders would likely increase genetic correlation estimates (Coop & Pickrell, 2016). Finally, there are technical and methodological artifacts (e.g. overlapping or related individuals) that may induce spurious genetic correlations in molecular genetic studies, which need to be ruled out.

More research is needed to determine the extent to which comorbidity, ascertainment bias, heterogeneity, diagnostic misclassification, and assortative mating inflate genetic correlations across psychiatric disorders and how much of these estimates are due to true pleiotropy. Even so, the possible biological interpretations of genetic correlations described above are hard to distinguish using the methods described in this review, as genetic correlations do not pinpoint the source of shared genetic risks. Some clues might be gained by partitioning heritability based on SNP functional category, position or frequency (Finucane *et al.* 2015), to try to better identify the source of the genetic correlations. Large-scale GWAS meta-analyses and sequencing studies are needed to find robust risk variants associated with multiple disorders.

After identifying specific genetic risk variants that correlate across disorders and considering the above possibilities, well-phenotyped samples and new methods will be needed to interpret the meaning of genetic correlations. Several newly developed methods have the potential to help with interpretation. The method ‘pairwise-GWAS’ aims to determine whether the effect sizes of variants associated with one trait are correlated with effect sizes of those variants for another trait and vice versa (Pickrell *et al.* 2016). Another method, BUHMBOX, aims to statistically differentiate between situations where there is sub-group heterogeneity (i.e. phenotype misclassification, different biological subtypes of a disorder, ascertainment bias or mediation) or whether there is true pleiotropy (Han *et al.* 2016).

## Implications for research and clinical practice

Despite moderate to high degrees of genetic correlation between some pairs of phenotypes, unique genetic factors are also likely to be important, as illustrated in Fig. 1*b*. This unique genetic risk is associated with important clinical distinctions that exist between disorders and also between disorders and continuous traits. For example, certain medications are effective for one disorder (e.g. stimulants for ADHD), but do not impact the symptoms of other disorders (Thapar *et al*. 2017). Also, in the absence of severe impairment resulting from symptoms, the cost-benefit ratio of treatment needs to be considered. Since most genetic correlations are below 1, more insights into the meaning of these correlations are required before clinical practice can be advanced.

The assumption that there is some true sharing of genetic risks has already led to insights into the genetic architecture and biology of psychiatric disorders through combining phenotypes in joint analyses to boost statistical power. For example, a joint GWAS analysis of five psychiatric disorders led to a more powerful approach for identifying genetic variants associated with psychiatric disorders (Cross-Disorder Group of the PGC, 2013*b*). Similarly, using the results of a GWAS of multiple psychiatric disorders can substantially increase the accuracy of PRS analyses (Maier *et al.* 2015). Also, a literature review of genetic sequencing studies of several childhood-onset neurodevelopmental disorders has shown the power of pooling information on multiple phenotypes to identify more robust genes implicated in neurodevelopmental disorders (Gonzalez-Mantilla *et al.* 2016). Gene discovery studies meta-analyzing GWAS of a clinical disorder with GWAS of population traits can benefit from substantially increased power to detect common variants, as can be seen for example for MDD and ADHD (Direk *et al.* 2016; Demontis *et al.* 2017). Understanding the nature and degree of shared genetic risks across psychiatric phenotypes will be essential to most effectively using this observation for future research into the genetic architecture of these disorders.

One important limitation of existing molecular genetic studies is that for many psychiatric disorders, sample sizes are still relatively small and analyses are limited in statistical power. PRS studies, in particular, tend to find low effect sizes. As larger and more reliable genetic samples become available in the future, it will be possible to better determine the degree and source of shared genetic risks across psychiatric phenotypes.

## Conclusion

Emerging evidence from twin and molecular genetic studies suggests that some genetic risk is shared between diagnosed disorders and variation in psychiatric traits in the population for certain disorders (e.g. ADHD) and across different psychiatric diagnoses (e.g. schizophrenia and BD). More research is needed to investigate the degree of genetic correlation across disorders and traits for other psychiatric phenotypes (e.g. anxiety or BD) and across pairs of different disorders (e.g. anorexia and OCD). Future research should then aim to identify specific genetic loci that are driving any genetic correlations and determine the nature of such correlations. However, recent insights into the genetic architectures of psychiatric disorders are already pointing towards new avenues for further research into the biology of these complex disorders.
